# Supplementary material for: A UMLS-based spell checker for natural language processing in vaccine safety
Source: BMC Med Inform Decis Mak. 2007 Feb 12;7:3. doi: 10.1186/1472-6947-7-3 (PMC1805499; doi:10.1186/1472-6947-7-3)
Supplement: Additional file 13 — RAP application directory. Sets up directory for RAP (RDF application for PHP) source code [file 1472-6947-7-3-S13.gz › rap/doc/phpdoc/packages.html]

xml version="1.0" encoding="iso-8859-1"?


|  |  |  |
| --- | --- | --- |
| RDF API Home \| Documentation \| PHPDoc | Packages  model dataset infModel ontModel rdql resModel syntax utility | RAP - Rdf API for PHP |
